# Protective Effects of Astragalus Polysaccharide on Sepsis-Induced Acute Kidney Injury

**DOI:** 10.1155/2021/7178253

**Published:** 2021-01-26

**Authors:** Jie Sun, Shanzhai Wei, Yilai Zhang, Jia Li

**Affiliations:** Department of Nephrology, Shuyang Hospital Affiliated to Nanjing University of Traditional Chinese Medicine, China 223600

## Abstract

**Objective:**

To explore the protective roles of Astragalus polysaccharide (APS) on acute renal injury (AKI) induced by sepsis.

**Methods:**

Firstly, an animal model of sepsis-induced AKI was established by injecting lipopolysaccharide (LPS) into mice. The mice were pretreated with an intraperitoneal injection of 1, 3, and 5 mg/(kg·d) APS for 3 consecutive days. The severity of kidney injury was then scored by histopathological analysis, and the concentrations of serum urea nitrogen (BUN) and serum creatinine (SCr) and the levels of tumor necrosis factor *α* (TNF-*α*) and interleukin-1*β* (IL-1*β*) were determined as well. In in vitro experiments, lipopolysaccharide (LPS) was used to induce HK-2 cell injury to establish a sepsis-induced AKI cell model, and the cell counting kit-8 (CCK-8) method was performed to determine the cytotoxicity and appropriate experimental concentration of APS. Then, cells were divided into the control, LPS, and APS+LPS groups. Cell apoptosis and inflammation-related TNF-*α*, IL-1*β*, IL-6, and IL-8 were determined by flow cytometry and enzyme-linked immunosorbent assay (ELISA), respectively. The microscope was used to observe the morphological changes of cells, and the cell migration ability was measured by wound healing assay. RT-qPCR and Western blot assay were used to determine the mRNA and protein levels of apoptosis-related factors including caspase-3, caspase-9, Bax, and Bcl-2; endoplasmic reticulum stress- (ERS-) related biomarkers including C/EBP homologous protein (CHOP) and glucose-regulated protein78 (GRP78); and epithelial-mesenchymal transition- (EMT-) related biomarkers including E-cadherin, Snail, *α*-smooth muscle actin (*α*-SM*Α*), and Vimentin.

**Results:**

In vivo experiments in mice showed that APS can reverse LPS-induced kidney damage in a concentration-dependent manner (*P* < 0.05); the concentrations of BUN and Scr were increased (all *P* < 0.05); similarly, the levels of TNF-*α* and IL-1*β* were increased as well (all *P* < 0.05). In in vitro experiments, the results showed that LPS can significantly cause HK-2 cell damage and induce apoptosis, inflammation, ERS, and EMT. When APS concentration was in the range of 0-200 *μ*g/mL, it had no cytotoxicity in HK-2 cells, and 100 *μ*g/mL APS pretreatment could significantly mitigate the decrease of cell activity induced by LPS (*P* < 0.05). Compared with the LPS group, APS pretreatment could inhibit the expression of inflammatory factors including TNF-*α*, IL-1 *β*, IL-6, and IL-8 (all *P* < 0.05), reducing the number of apoptotic cells (*P* < 0.05), suppressing the expression of caspase-3, caspase-9, and Bax, but upregulating the expression levels of Bcl-2. In ERS, APS pretreatment inhibited LPS-induced upregulation of CHOP and GRP78. Moreover, in EMT, APS pretreatment could inhibit the morphological changes of cells, downregulate the migration, decrease the expression of EMT biomarkers, and inhibit the process of EMT.

**Conclusion:**

APS could alleviate sepsis-induced AKI by regulating inflammation, apoptosis, ERS, and EMT.

## 1. Introduction

Acute kidney injury used to be called acute kidney failure. It is a clinically serious disease caused by complicated factors such as surgery, multiple organ dysfunction, and coma. It could lead to a rapid decline in kidney function and kidney damage [1]. In China, at least 2.9 million adult AKI patients are hospitalized each year, about 40% of them die from AKI, and a considerable number of patients cannot fully recover [2]. One of the reasons which result in this situation is that clinicians lack sufficient clinical understanding of this disease and inappropriate diagnosis and treatment. Sepsis is a systemic inflammatory response syndrome caused by infection. The progressive exacerbation of sepsis can lead to multiple organ dysfunction, especially renal dysfunction. The incidence of AKI in ICU patients is 31.6%, of which 32.4% had sepsis [3]. The risk of death of AKI patients caused by sepsis is significantly increased, and some patients with sepsis-induced AKI may develop chronic kidney disease. Therefore, strengthening the prevention and treatment of patients with sepsis-induced AKI is of great significance. The pathogenesis of sepsis-induced AKI is closely related to abnormal renal hemodynamics, inflammatory injury, apoptosis, and adaptive response [4]. A major cause of sepsis AKI is lipopolysaccharide- (LPS-) mediated apoptosis of renal tubular epithelial cells [5]. LPS is a component of the outer membrane of Gram-negative bacteria and participates in the pathogenesis of sepsis-induced AKI [6]. Mariano et al. found *in vitro* that plasma extracted from patients with severe sepsis or septic shock can induce apoptosis and functional changes in renal tubular epithelial cells [7]. In addition, studies have shown that endoplasmic reticulum stress (ERS) is also involved in the pathological process of AKI [8, 9]. When there are abnormal damage factors in the human body, the ER cannot transport proteins normally and the protein accumulation in the ER can induce ERS. It is known that ERS can cause apoptosis and other reactions in cells [10]. However, there are few studies on AKI and ERS in sepsis, so it is urgent to explore. With the development of AKI, the severity of kidney injury is positively correlated with the degree of renal fibrosis. Studies have confirmed that renal tubular epithelial-mesenchymal transition (EMT) is one of the important mechanisms of renal fibrosis [11, 12]. However, the role of EMT in the progression of sepsis AKI has not yet been elucidated.

Astragalus is one of the traditional Chinese medicines, widely used in the treatment of various diseases, including kidney disease. Astragalus has a complex chemical composition, and its main active ingredients include astragaloside, flavonoids, and polysaccharides [13]. In the treatment of sepsis, studies have confirmed that Astragalus polysaccharides (APS) have anti-inflammatory, proliferative, and immune-regulating effects [14–16]. In the study of kidney disease, Ma et al. found that APS pretreatment can prevent cisplatin-induced AKI by reducing oxidative damage and resisting apoptosis [17]. Therefore, this study intends to first establish a sepsis AKI mouse model in vivo to study the protective and anti-inflammatory effects of APS on mouse kidneys and then establish the sepsis AKI cell model *in vitro* to study the effect of APS on inflammation, apoptosis, ERS, and EMT of sepsis AKI and to provide the possibility of finding drugs that can effectively treat sepsis-induced AKI.

## 2. Material and Methods

### 2.1. In Vivo Experiment

#### 2.1.1. Construction and Grouping of the Sepsis Mouse Model

C57BL/6 mice (22-26 g, SFP grade) were purchased from Changzhou Cavens Animal Laboratory Co., Ltd., China. All experimental operations involving mice in this study have been approved by the ethics committee of our hospital. 60 mice were randomly divided into 3 groups: control group (control, *n* = 15), LPS group (*n* = 15), and APS+LPS group (*n* = 15). The mice were then injected intraperitoneally with 1, 3, and 5 mg/(kg·d) APS for 3 consecutive days. After the last administration, all mice except the control group were intraperitoneally injected with LPS 10 mg/kg. The mice in the control group were intraperitoneally injected with the same amount of saline.

#### 2.1.2. Histopathological Examination

The mice were killed 12 hours after LPS treatment, and the right kidney was collected. The kidney tissue was fixed with 10% paraformaldehyde and embedded in paraffin, sectioned, and stained with hematoxylin and eosin for morphological examination. A blind method was used to allow the observer to give a semiquantitative score on the damage of each mouse's kidney. The specific scoring rules are as follows: the percentage scores showing cell necrosis, the disappearance of brush borders, interstitial edema, vacuolation, and tubule expansion where 0 = 0%, 1 = 0–20%, 2 = 20%–50%, 3 = 50%–70%, and 4 = 70% or more. At least 10 fields of view in the kidney tissue of each mouse were observed [18].

#### 2.1.3. The Effect of LPS on Mouse Renal Function and Levels of Inflammatory Cytokine Determined by Biochemical Measurements

The concentration of blood urea nitrogen (BUN) and serum creatinine (SCr) in mice was measured with SpectraMax-M2 multimode plate reader, which were expressed in mmol/L and *μ*mol/L, respectively.

After 12 hours of LPS treatment, the mice were sacrificed, and the left kidney was quickly removed. The kidney tissue was then ground with a homogenizer in phosphate-buffered saline (pH 7.4, *w*/*v*; 1 g tissue and 9 mL PBS). After centrifugation at 4°C with 10,000 g for 10 min, the ELISA kit was then used to measure the level of TNF-*α* and IL-1*β* in the supernatant according to the manufacturer's instructions.

### 2.2. In Vitro Experiments

#### 2.2.1. Cell Culture and Construction of Sepsis AKI Cell Model

The human renal proximal tubular epithelial cell line (HK-2) was purchased from ATCC (USA). The cells were cultured in F-12 (DMEM/F-12, Gibco, United States) medium containing 10% fetal bovine serum (Gibco, United States), 100 U/mL penicillin, and 100 mg/mL streptomycin and placed in a humidified incubator with 5% CO_2_ at 37°C. Before modeling, cells with 90% confluence were digested and passaged with EDTA-trypsin digestion solution (Solarbio, China), and then, HK-2 cells were inoculated in the culture vessel and cultured until the next day. After that, the original culture solution was removed, and the cell culture medium with an LPS concentration of 10 ng/mL was added. 24 hours later, HK-2 cells were collected for subsequent experiments. For the control group, PBS was used instead of LPS. For the specific modeling method, see reference [18].

#### 2.2.2. Cell Counting Kit-8 (CCK-8) Method to Determine Cell Activity

The effect of LPS and APS on the activity of HK-2 cells was determined by the CCK-8 (Dojindo, Japan) method. HK-2 cells were seeded in 96-well plates at a density of 1 × 10^4^ cells/well and cultured overnight. LPS toxicity test: the original cell culture fluid was removed the next day, and then, the LPS group was added with a cell culture fluid containing 10 ng/mL LPS, while the control group was added with a cell culture fluid containing 10 ng/mL PBS, after which cells were incubated for 24 hours. APS toxicity test: first, APS (Boster Biology Co., China) was dissolved in cell culture fluid, and APS cell culture fluid with different concentrations (0, 50, 100, 150, 200, 250, and 300 *μ*g/mL) was prepared. The next day after cell inoculation, HK-2 cells were cultured with a culture medium containing different concentrations of APS for 3 hours. The effect of APS on LPS-induced cytotoxicity: the next day after cell inoculation, HK-2 cells were cultured with different concentrations (0, 50, 100, 150, and 200 *μ*g/mL) of APS culture medium for 3 hours. After that, the culture was removed, and the preprepared cell culture medium containing 10 ng/mL LPS was added and incubated for 24 hours. After the incubation, 10 *μ*L CCK-8 was added to each well, and the cell was put back into the incubator to incubate for another 2 hours. Thereafter, the multifunctional microplate reader (PerkinElmer Inc., USA) was used to measure the absorbance at 450 nm (OD). A blank group with a culture medium was used as a background control. The calculation formula of relative cell activity is as follows: relative cell activity (%) = (OD_sample_ − OD450_blank_)/(OD450_control_ − OD590_blank_) × 100.

#### 2.2.3. Enzyme-Linked Immunosorbent Assay to Determine the Expression Levels of TNF-*α*, IL-1*β*, IL-6, and IL-8

HK-2 cells were inoculated in a 24-well plate at a cell density of 2 × 10^5^ cells/well and incubated overnight. The original culture medium was removed the next day; for the control group: cell culture fluid containing 0 *μ*g/mL APS was added and incubated for 3 hours, then the original culture fluid was removed, and culture fluid with PBS instead of LPS was added to incubate for another 24 hours. For the LPS group: cell culture fluid containing 0 *μ*g/mL APS was added and incubated for 3 hours, then the original culture fluid was removed, and culture fluid with 10 ng/mL LPS was added to incubate for another 24 hours. For the APS+LPS group, cell culture medium containing 100 *μ*g/mL APS was added to the culture for 3 hours, then the original culture medium was removed, and culture medium containing 10 ng/mL LPS was added to incubate for another 24 hours. After culturing, the cell culture fluid was collected; TNF-*α*, IL-1*β*, IL-6, and IL-8 ELISA kits were used for the determination of the corresponding inflammatory factors (the above kits are all purchased from R&D Systems, USA). Finally, the microplate reader was used to measure the absorbance at 450 nm, and then, a standard curve was drawn and the concentration was calculated.

#### 2.2.4. Light Microscope Observation of Cell Morphology Changes

Cell processing steps are the same as the method in Section 2.2.3. After the treatment, the cell morphology was observed under an inverted microscope (Olympus, Japan).

#### 2.2.5. Scratch Experiment to Detect Cell Migration Ability

Cell processing steps are the same as the method in Section 2.2.3. After the treatment, the cells were digested with EDTA-trypsin digestion solution, reseeded in 6-well plates, and cultured to the next day. After that, a sterile pipette tip was used to draw parallel lines on the bottom of the wells of the cell culture plate, and then, the PBS was used to rinse off the cells and the normal cell culture medium was used for culturing. Finally, photographs of scratch width (*D*) were taken under an inverted microscope at 0 h and 24 hours. Relative cell migration capacity (%) = (*D*_sample−0 h_ − *D*_sample−24 h_)/(*D*_control−0 h_ − *D*_control−24 h_) × 100.

#### 2.2.6. Determination of Apoptosis by Flow Cytometry

Cell processing steps are the same as the method in Section 2.2.3. Annexin V-FITC/PI apoptosis kit (Shengong, China) was used to detect the level of apoptosis. After the treatment, the cells were digested with trypsin digestion solution without EDTA, and cells were collected after digestion. Then, the cells were washed with prechilled PBS and centrifuged at 3000 rpm at room temperature to keep the pellet, which was repeated twice. After that, 400 *μ*L of binding buffer was added to resuspend the cell pellet, and 15 *μ*L Annexin V-FITC and 5 *μ*L PI were added at room temperature, avoiding light to costain for 15 min, which was then used to detect the apoptosis by flow cytometry (Becton Dickinson, USA).

#### 2.2.7. Real-Time Quantitative Polymerase Chain Reaction (RT-qPCR) to Detect mRNA Expression

Cell processing steps are the same as the method in Section 2.2.3. After the treatment, TRIzol (Life Technologies, USA) was used to extract the total RNA of HK-2 cells, and 1 *μ*g of RNA was taken to obtain cDNA by using RevertAid First Strand cDNA Synthesis Kit (Thermo Scientific, USA). Then, One Step SYBR® PrimeScript® PLUS RT-RNA PCR Kit (Takara, Japan) was used to relatively quantify the target gene, and the 2^−*ΔΔ*Ct^ method was used to calculate the expression level of the target gene relative to the housekeeping gene GAPDH. Primer sequences are shown in Table 1.

#### 2.2.8. Detection of Protein Expression by Western Blot

Cell processing steps are the same as the method in Section 2.2.3. After the treatment, the culture solution was removed and the cells were washed twice with prechilled PBS. Then, 0.2 mL of strong RIPA lysate (Biyuntian, China) was added, and the cell lysate was collected using a cell scraper and centrifuged with 12,000 × g at 4°C for 15 min to leave the supernatant in a clean 1.5 mL centrifuge tube. After that, the tube was placed on a 98°C heater for 10 min to denature the cells, and the BCA protein concentration determination kit (Takara, Japan) was used to determine the protein concentration. Finally, samples were stored in the refrigerator at -20°C. The appropriate concentration of SDS-PAGE electrophoresis gel corresponding to the molecular weight of the target protein was prepared, and the volume required to load 30 *μ*g sample per well was calculated according to the determined protein concentration. After sample loading and electrophoresing, the protein on the electrophoresis gel was transferred to the PVDF membrane (Millipore, USA) and incubated on a shaker with 180 rpm/min at room temperature for 2 hours. After incubating, TBST (Biyuntian, China) was used to wash the membrane twice, 5 min each time. After washing, the primary antibodies of the target protein including Bax (ab32503, Abcam, United Kingdom), Bcl-2 (ab182858, Abcam, United Kingdom), caspase-3 (ab32150, Abcam, United Kingdom), cleaved caspase-3 (ab32042, Abcam, China), caspase-9 (ab32539, Abcam, United Kingdom), cleaved caspase-9 (ab2324, Abcam, China), CHOP (ab11419, United Kingdom), GRP78 (ab191023, United Kingdom), E-cadherin (#14472, Cell Signaling Technology, United States), *α*-SMA (#48938, Cell Signaling Technology, United States), Snail (#3879, Cell Signaling Technology, United States), Vimentin (#12826, Cell Signaling Technology, United States), and GAPDH (ab8245, Abcam, United Kingdom) were used to incubate the PVDF membrane in a shaker with 180 rpm/min at 4°C overnight. The primary antibody was recovered the next day, and the membrane was washed twice with TBST and then incubated with a corresponding secondary antibody in a shaker with 180 rpm/min at room temperature for 2 hours. Thereafter, the membrane was washed twice with TBST, and an ECL luminescence developer (Biyuntian, China) was added to the membrane, which was then placed in an imaging system (ImageQuant program) for exposure and image development.

### 2.3. Statistical Analysis

The experiments in this study were independently repeated at least 3 times. The measurement data generated by the experiment were expressed as mean ± standard deviation. The Student *t*-test was used for comparative analysis within the group, and the one-way analysis of variance was used for comparative analysis between the groups. *P* < 0.05 means the difference is statistically significant.

## 3. Results

### 3.1. The Effects of APS on LPS-Induced AKI

Histopathological changes are a direct indication of AKI. In the control group, the kidney tissue had a normal tubular structure. And LPS can significantly cause renal histopathological changes, including renal tubular epithelial cell edema, glomerular atrophy, renal capsule cavity expansion, renal tubular structure destruction, focal necrosis, and the collapse of epithelial cells, as well as renal interstitial epithelial cell edema. However, APS treatment (1, 3, and 5 mg/(kg·d)) can significantly reduce LPS-induced epithelial atrophy and necrosis, as well as varying degrees of interstitial edema. As shown in Table 2, the results of renal pathological damage score were consistent with our observations, that is, compared with the control group, LPS induction can significantly increase the damage score of the kidney tissue (*P* < 0.05), whereas the administration of APS pretreatment can significantly reverse the AKI damage (*P* < 0.05) and the protective effects was in a concentration-dependent manner (*P* < 0.05).

### 3.2. The Effects of APS on AKI Renal Function Damage Caused by LPS

**Figure 1:**
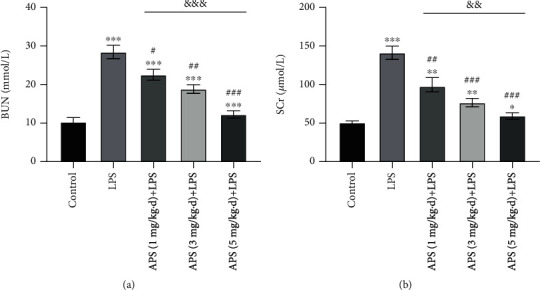
Effects of APS on the concentration of serum (a) BUN and (b) SCr. ^∗^*P* < 0.05, ^∗∗^*P* < 0.01, and ^∗∗∗^*P* < 0.001 versus the control group; ^#^*P* < 0.05, ^###^*P* < 0.01, and ^###^*P* < 0.001 versus the LPS group; ^&&^*P* < 0.01 and ^&&&^*P* < 0.001 versus different concentrations of APS treatment in groups.

As shown in Figure 1, by measuring the serum biochemical indicators, it was found that the levels of SCr and BUN in the LPS-induced group were significantly higher than those in the control group (all *P* < 0.05), while the levels of BUN and SCr in the APS treatment group were dose-dependently decreased (all *P* < 0.05).

### 3.3. The Effects of APS on the Levels of Inflammatory Cytokines in LPS-Induced AKI

**Figure 2:**
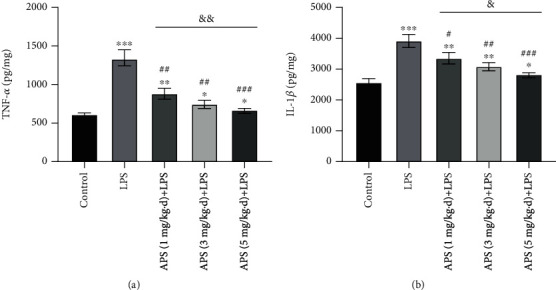
Effects of APS on (a) TNF-*α* and (b) IL-1*β* in kidney tissue. ^∗^*P* < 0.05, ^∗∗^*P* < 0.01, and ^∗∗∗^*P* < 0.001 versus the control group; ^#^*P* < 0.05, ^###^*P* < 0.01, and ^###^*P* < 0.001 versus the LPS group; ^&^*P* < 0.05 and ^&&^*P* < 0.01 versus different concentrations of APS treatment in groups.

To explore the proinflammatory molecules produced after LPS-induced kidney injury, the cytokine levels of TNF-*α* and IL-1*β* in kidney tissue were measured. As shown in Figure 2, the levels of TNF-*α* and IL-1*β* in LPS-treated mice were significantly increased compared to that of control (both *P* < 0.05), while APS pretreatment significantly reduced the level of TNF-*α* and IL-1*β* induced by LPS in a dose-dependent manner (all *P* < 0.05).

### 3.4. LPS Induces HK-2 Cell Damage

**Figure 3:**
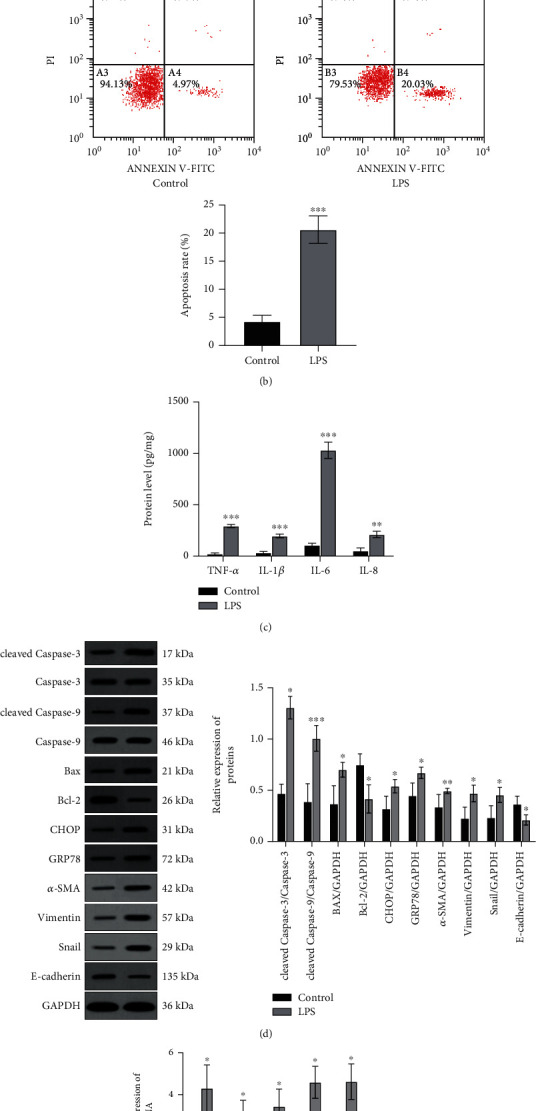
LPS induced HK-2 cell damage. (a) CCK-8 assays were used to detect cell viability; (b) flow cytometry was used for assessment of cell apoptosis; (c) ELISA was performed for estimation of TNF-*α*, IL-1*β*, IL-6, and IL-8 expression and secretion. (d) Western blot was performed to determine the protein levels of caspase-3, caspase-9, Bax, Bcl-2, CHOP, GRP78, E-cadherin, *α*-SMA, Snail, and Vimentin, where GAPDH was used as an internal control; (e) RT-qPCR assay was used for evaluation of the mRNA levels of caspase-3, caspase-9, Bax, Bcl-2, CHOP, GRP78, E-cadherin, *α*-SMA, Snail, and Vimentin, in which GAPDH was used as an internal control gene to calculate the relative expression of the target genes. ^∗^*P* < 0.05, ^∗∗^*P* < 0.01, and ^∗∗∗^*P* < 0.001 versus the control group.

In this study, the HK-2 cells were first treated with 10 ng/mL LPS, and the effects of LPS on cell activity, inflammatory response, apoptosis, ERS, and EMT were measured. As shown in Figures 3(a) and 3(b), compared with the control group, LPS can significantly reduce HK-2 cell activity (*P* < 0.05) and increase the number of apoptotic cells (*P* < 0.05). As shown in Figure 3(c), it was also found that LPS can significantly promote the production of inflammation-related factors TNF-*α*, IL-1*β*, IL-6, and IL-8 in HK-2 cells (all *P* < 0.05). Besides, as shown in Figures 3(d) and 3(e), in the process of LPS-induced apoptosis of HK-2 cells, LPS can promote the expression of apoptosis-related proteins caspase-3, caspase-9, and Bax (all *P* < 0.05), but inhibit the expression of antiapoptotic protein Bcl-2 (*P* < 0.05). Moreover, LPS treatment can also increase the expression of ERS markers CHOP and GRP78 and EMT markers *α*-SMA, Snail, and Vimentin (all *P* < 0.05), while it can decrease the expression of E-cadherin (*P* < 0.05). It is suggested that LPS-induced HK-2 cell damage is related to ERS and EMT in addition to inducing apoptosis and inflammatory response.

### 3.5. APS Pretreatment Reduces LPS-Induced HK-2 Cell Damage

**Figure 4:**
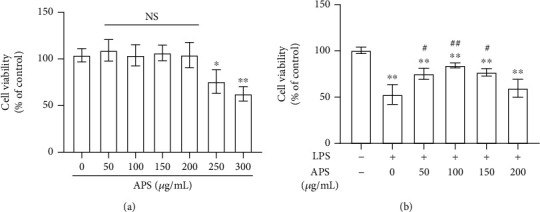
APS pretreatment protected HK-2 cells from LPS exposure. (a) Cell viability of HK-2 cells treated with APS at different concentrations (0, 50, 100, 150, 200, 250, and 300 *μ*g/mL) for 3 h; (b) cell viability of LPS treatment for 24 h in HK-2 cells after pretreated with various concentrations (0, 50, 100, 150, and 200 *μ*g/mL) of APS. Compared with the control group, ^∗^*P* < 0.05 and ^∗∗^*P* < 0.01; compared with the LPS group, ^#^*P* < 0.05, ^##^*P* < 0.01, and ^###^*P* < 0.001.

The effect of APS on HK-2 cell activity was measured. As shown in Figure 4(a), different doses of APS (50, 100, 150, and 200 *μ*g/mL) had no cytotoxic effect (all *P* > 0.05). However, when reaching 250 and 300 *μ*g/mL, compared with the control group (0 *μ*g/mL), HK-2 cell activity was significantly reduced (both *P* < 0.05). In the follow-up study of the effect of APS pretreatment on LPS-induced HK-2 cell damage, HK-2 cells were first pretreated with 0-200 *μ*g/mL APS for 3 hours and then treated with 10 ng/mL LPS for 24 hours. As shown in Figure 4(b), 100 *μ*g/mL APS had the greatest protective effect on LPS-induced HK-2 cell damage (*P* < 0.05). Therefore, 100 *μ*g/mL APS was used for subsequent experimental pretreatment.

### 3.6. APS Pretreatment Inhibits the Inflammatory Response in Sepsis AKI Cell Model

**Figure 5:**
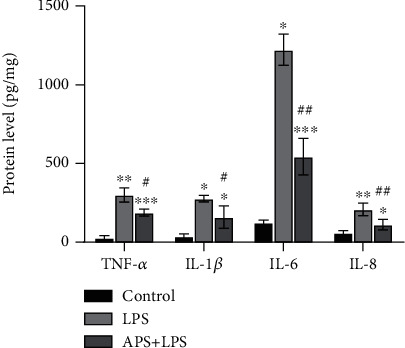
APS alleviated HK-2 cell inflammatory injury induced by LPS. Protein levels of inflammatory factors (TNF-*α*, IL-1*β*, IL-6, and IL-8) of HK-2 cells in the control, LPS, and APS+LPS groups were detected by ELISA. Compared with the control group, ^∗^*P* < 0.05, ^∗∗^*P* < 0.01, and ^∗∗∗^*P* < 0.001; compared with the LPS group, ^#^*P* < 0.05 and ^##^*P* < 0.01.

As shown in Figure 5, compared with the significantly increased protein levels of inflammatory factors TNF-*α*, IL-1*β*, IL-6, and IL-8 in the LPS group (all *P* < 0.05), APS pretreatment can significantly inhibit the above inflammatory factor protein expression level (all *P* < 0.05), therefore inhibiting the occurrence of inflammation.

### 3.7. APS Pretreatment Inhibits the Apoptotic Response in Sepsis AKI Cell Model

**Figure 6:**
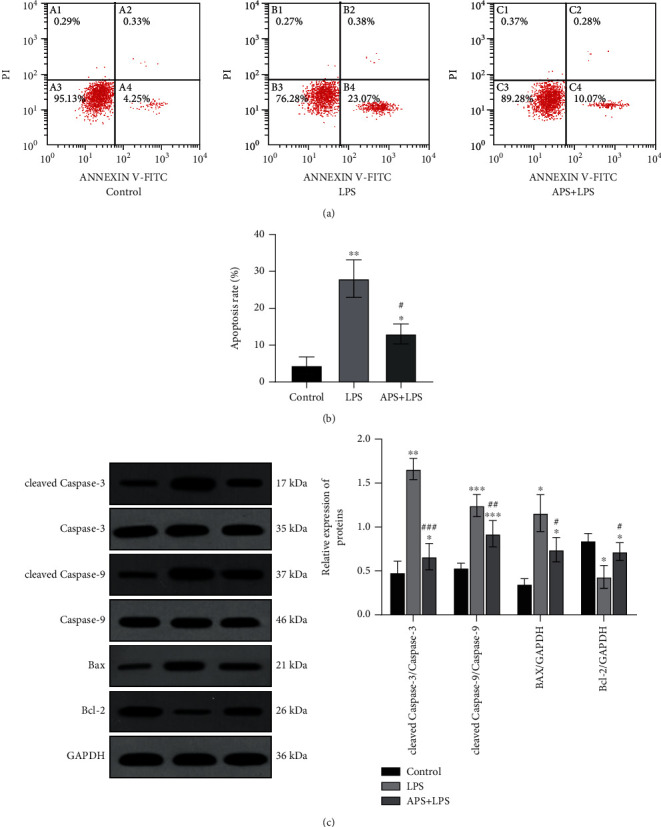
APS ameliorates LPS-induced apoptosis in HK-2 cells. (a) The apoptosis rate of cells with LPS, APS, and LPS+APS in HK-2 cells by flow cytometry; (b) the levels of apoptosis-related protein including cleaved caspase-3, caspase-3, cleaved caspase-9, caspase-9, Bax, and Bcl-2 in cells treated with LPS, APS, and LPS+APS in HK-2 cells determined by Western blot, and GAPDH was used as an internal control protein to calculate the relative expression of proteins. Compared with the control group, ^∗^*P* < 0.05, ^∗∗^*P* < 0.01, and ^∗∗∗^*P* < 0.001; compared with the LPS group, ^#^*P* < 0.05, ^##^*P* < 0.01, and ^###^*P* < 0.001.

As shown in Figure 6(a), compared with the significantly increased number of apoptotic cells in the LPS group (*P* < 0.05), APS pretreatment can significantly reduce the number of apoptotic cells induced by LPS (*P* < 0.05) and inhibit the occurrence of apoptotic reactions. In addition, as shown in Figure 6(b), Western blot detection found that compared with the control group, the expression of the apoptotic proteins caspase-3, caspase-9, and Bax in the LPS group was increased (all *P* < 0.05), while the antiapoptotic protein Bcl-2 protein expression was decreased (*P* < 0.05), and the addition of APS pretreatment can reverse the above protein expression changes (all *P* < 0.05), which are consistent with the results of flow cytometry.

### 3.8. APS Pretreatment Inhibits ERS Response in Sepsis AKI Cell Model

**Figure 7:**
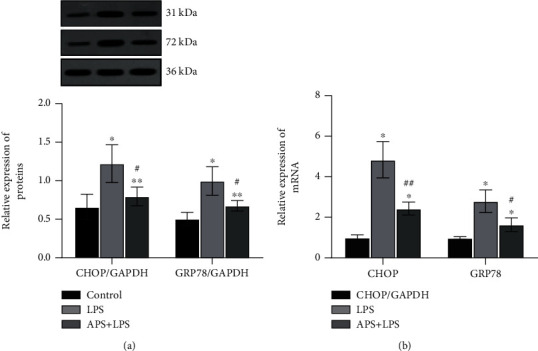
APS suppressed LPS-induced ERS in HK-2 cells. (a) Western blot was used to detect the protein levels of CHOP and GRP78, and GAPDH was used to be an internal control; (b) RT-qPCR assay was used for evaluation of the mRNA levels of CHOP and GRP78, and GAPDH was used as an internal control gene to calculate the relative expression of the target genes. Compared with the control group, ^∗^*P* < 0.05 and ^∗∗^*P* < 0.01; compared with the LPS group, ^#^*P* < 0.05 and ^##^*P* < 0.01.

As shown in Figures 7(a) and 7(b), compared with the significantly increased protein and mRNA levels of CHOP and GRP78 in the LPS group (both *P* < 0.05), APS pretreatment significantly decreased the expression levels of CHOP and GRP78 (both *P* < 0.05), which indicates that APS can inhibit the occurrence of ERS reaction.

### 3.9. APS Pretreatment Inhibits EMT in Sepsis AKI Cell Model

**Figure 8:**
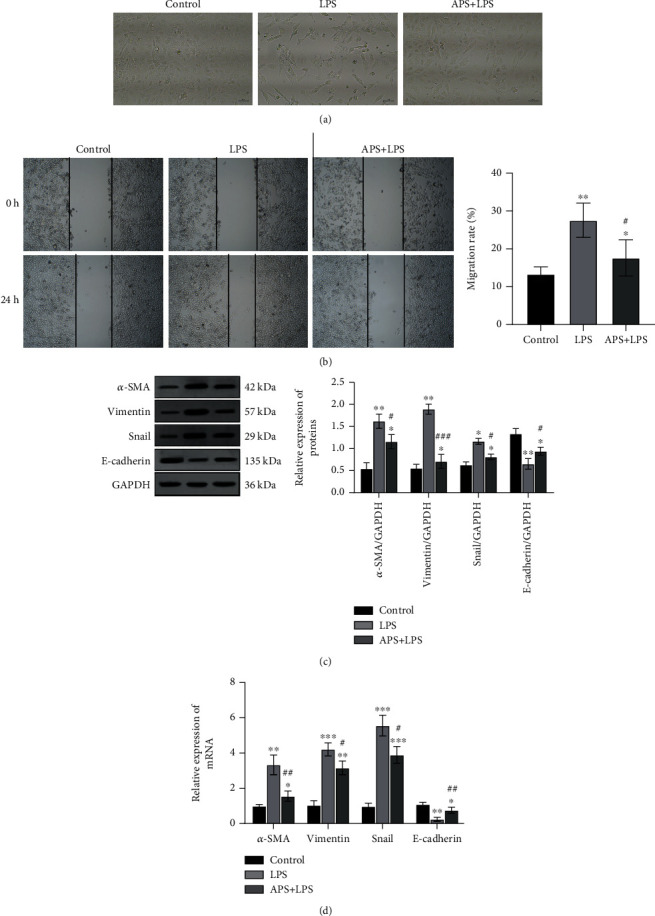
APS inhibited LPS-induced EMT in HK-2 cells. (a) Morphological changes of cells observed by light microscopy; (b) wound healing assay was used to detect cell migration; (c) Western blot was performed to detect the protein levels of E-cadherin, *α*-SMA, Snail, and Vimentin, and GAPDH was used to be an internal control; (d) RT-qPCR assay was used for evaluation of the mRNA levels of E-cadherin, *α*-SMA, Snail, and Vimentin, and GAPDH was used as an internal control gene to calculate the relative expression of the target genes. Compared with the control group, ^∗^*P* < 0.05, ^∗∗^*P* < 0.01, and ^∗∗∗^*P* < 0.001; compared with the LPS group, ^#^*P* < 0.05, ^##^*P* < 0.01, and ^###^*P* < 0.001.

As shown in Figure 8(a), compared with HK-2 cells in the control group, LPS induced morphological remodeling of the cells, which showed increased branching, increased intercellular space, and spindle shape, suggesting that occurrence of EMT in LPS-induced HK-2 cells, and APS pretreatment can reduce the above morphological changes. In this study, scratch experiments were used to confirm cell migration ability as a functional change of EMT. As shown in Figure 8(b), compared with normal HK-2 cells, LPS induced cell migration ability, while APS pretreatment can reduce cell migration ability (*P* < 0.05). As shown in Figures 8(c) and 8(d), compared with the LPS group, APS pretreatment can significantly inhibit the protein and mRNA expression levels of *α*-SMA, Vimentin, and Snail (all *P* < 0.05), but promote the expression of E-cadherin (all *P* < 0.05).

## 4. Discussion

AKI is an acute disease that seriously threatens the life and health of patients. The mortality rate of AKI patients caused by sepsis is more than 70%, which is significantly higher than that of sepsis patients without AKI [19]. It has been reported that sepsis-induced AKI exhibits significant inflammatory response-mediated damage and is accompanied by significant apoptosis [20]. In this study, we first confirmed through experiments in mice that APS can inhibit AKI damage caused by LPS, can reduce the concentrations of BUN and SCr, and can reduce the levels of TNF-*α* and IL-1*β*. In terms of the in vitro experiments, we first applied LPS to induce sepsis damage in HK-2 cells, measured cell proliferation, inflammation, apoptosis, ERS, and EMT, and then explored whether APS plays a protective role in LPS-damaged HK-2 cells. Interestingly, the experimental results show that APS can inhibit LPS-induced inflammatory damage by reducing the level of proinflammatory factors. In addition, we also confirmed that APS can regulate the apoptosis, ERS, and EMT responses of HK-2 cells treated with LPS.

APS is a water-soluble heteropolysaccharide extracted from the dry roots of Astragalus. It is known that Astragalus has a history of more than 2000 years in the field of Chinese medicine in China. It has been proven by research and clinical evidence that it has various pharmacological activities such as anti-inflammatory, antitumor, antidiabetic, antiviral, hepatoprotective, antiatherosclerosis, hematopoiesis, and neuroprotection [13]. In kidney disease, APS has been found to prevent cisplatin-induced AKI by reducing oxidative damage and antiapoptosis [17] and preventing kidney stones by repairing cells, inhibiting adhesion, and promoting endocytosis [20]. Studies have reported that the protective effect of APS on organs may be related to its anti-inflammatory activity [13, 17].

BUN and SCr are important indicators for the severity of the renal injury and are often used to evaluate renal function. Lian et al. found that in a rat model of chronic renal failure, APS can reduce the concentration of BUN and SCr [21]. In this study, we found through in vivo experiments that in the LPS-induced mouse AKI model, APS treatment can significantly reduce the degree of kidney damage as well as decrease the levels of damage indicators such as BUN and SCr. Therefore, this study is the first one to our best knowledge to confirm the protective effect of APS in the AKI model. Besides, it is known that TNF-*α*, IL-1*β*, IL-6, and IL-8 are important inflammatory cytokines that participate in the body's immune response and subsequent inflammation. Similar to the results of BUN and SCr, we also confirmed that APS pretreatment can significantly reduce the levels of TNF-*α* and IL-1*β* in mouse kidneys induced by LPS, suggesting that APS can inhibit the inflammatory response caused by LPS, further demonstrating its protective effect.

In cell experiments, Oztas et al. found that LPS can increase the expression of IL-1*β* and IL-6 in HK-2 cells [22]. In this study, by treating HK-2 cells with 10 ng/mL LPS, it was found that LPS can induce the expression increase of TNF-*α*, IL-1*β*, IL-6, and IL-8, confirming that LPS can induce HK-2 inflammation damage to cells. Subsequently, by using a certain concentration of APS for pretreatment before LPS exposure, it was found that this pretreatment can effectively reduce the levels of the above inflammatory cytokines, which suggest that APS can reduce the inflammation damage induced by LPS by reducing the expression of proinflammatory cytokines.

In the occurrence and development of AKI, inflammatory infiltration can induce apoptosis, which in turn causes renal tubular epithelial cell dysfunction, which is also an important feature of AKI [23, 24]. More and more evidence showed that the main mechanism of renal tubular cell apoptosis is the activation of proapoptotic factors caspase-3, caspase-9, and Bax and the inhibition of antiapoptotic factor Bcl-2. Therefore, in addition to using flow cytometry to detect apoptosis, this study also observed the regulatory effect of APS on the above-mentioned proapoptotic and antiapoptotic factors. Flow cytometry results showed that APS pretreatment can significantly reduce the number of LPS-induced apoptotic cells. Moreover, APS pretreatment can also inhibit the expression of proapoptotic factors including caspase-3, caspase-9, and Bax in the AKI cell model induced by LPS. In contrast, the expression of antiapoptotic factor Bcl-2 was increased after APS pretreatment. This result suggests that APS can inhibit the apoptosis of renal tubular epithelial cells in the pathogenesis of LPS-induced sepsis-induced AKI.

In recent years, research on the regulatory mechanism of ERS in the pathogenesis of sepsis has increasingly attracted people's attention [25]. The endoplasmic reticulum response is a complex intracellular reaction in the endoplasmic reticulum, which can be triggered by certain physiological and pathological conditions, such as ischemia-reperfusion injury and inflammation [26]. It has been suggested that sepsis is related to endoplasmic reticulum activation, that is, the disorder of intracellular homeostasis caused by sepsis can destroy the homeostasis of the endoplasmic reticulum, thereby causing ERS [25, 27]. Previous studies have found that APS can inhibit ERS by regulating the miR-204/SIRT1 axis of retinal pigment epithelial cells [28]. The results of this study show that LPS can induce ERS in HK-2 cells and increase the expression of ERS-related markers CHOP and GRP78, while APS pretreatment inhibited the increase of mRNA and protein expression of CHOP and GRP78 in HK-2 cells. This result indicated that APS has an inhibitory effect on renal tubular epithelial cell ERS in the pathogenesis of LPS-induced sepsis AKI.

A large number of studies have confirmed that EMT is one of the important mechanisms of kidney fibrosis [11, 12] and is also the key to the progression of AKI to chronic kidney disease and renal failure. Clarifying the progress of AKI and preventing it is very important for EMT research. Typical markers for EMT in epithelial cells include *α*-SMA, E-cadherin, and Vimentin [29]. Increased expression of *α*-SMA and decreased expression of E-cadherin are one of the characteristic signs of EMT [30]. In this study, it was first observed that LPS exposure can induce HK-2 cells to undergo morphological changes, specifically showing increased branching and increased intercellular space. The scratch experiments showed increased cell migration ability and promoted expression of *α*-SMA and Vimentin HK-2 cell, but decreased expression of E-cadherin. The above results suggest that LPS induces EMT in HK-2 cells. Subsequently, by performing APS pretreatment, it was found that APS can reverse LPS-induced epithelial mesenchymalization of HK-2 cells and reduce cell migration ability. In addition, APS may inhibit LPS-induced increase in *α*-SMA and Vimentin expression, but increase the expression of E-cadherin, suggesting that APS may play a role in inhibiting EMT in sepsis-induced AKI.

## 5. Conclusion

In summary, this study found that APS can play a protective regulatory role in AKI caused by sepsis, that is, APS can reduce inflammation induced by sepsis by regulating inflammation, apoptosis, ERS, and EMT. An in-depth understanding of this mechanism is expected to provide direction for the future treatment of AKI.

## Figures and Tables

**Table 1:** Primer sequence.

Gene	Forward (5′ → 3′)	Reverse (5′ → 3′)
CHOP	GGAAACAGAGTGGTCATTCCC	CTGCTTGAGCCGTTCATTCTC
GRP78	CATCACGCCGTCCTATGTCG	CGTCAAAGACCGTGTTCTCG
E-cadherin	CGAGAGCTACACGTTCACGG	GGGTGTCGAGGGAAAAATAGG
Snail	TCGGAAGCCTAACTACAGCGA	AGATGAGCATTGGCAGCGAG
*α*-SMA	GGCATTCACGAGACCACCTAC	CGACATGACGTTGTTGGCATAC
Vimentin	GACGCCATCAACACCGAGTT	CTTTGTCGTTGGTTAGCTGGT
GAPDH	GGAGCGAGATCCCTCCAAAAT	GGCTGTTGTCATACTTCTCATGG

**Table 2:** Pathological score of representative kidney samples of each group.

Group	Kidney injury score
Control	0
LPS	4.10 ± 0.35^∗∗∗^
APS (1 mg/kg·d) + LPS	3.07 ± 0.18^∗∗∗^^#&&&^
APS (3 mg/kg·d) + LPS	2.40 ± 0.10^∗∗^^##&&&^
APS (5 mg/kg·d) + LPS	1.07 ± 0.13^∗∗^^###&&&^

Note: compared with the control group, ^∗∗^*P* < 0.01 and ^∗∗∗^*P* < 0.001; compared with the LPS group, ^#^*P* < 0.05, ^##^*P* < 0.01, and ^###^*P* < 0.001; compared with different concentrations of APS treatment groups, ^&&&^*P* < 0.001.

## Data Availability

All data generated or analyzed during this study are included in this published article.
